# Precoder and Decoder Co-Designs for Radar and Communication Spectrum Sharing †

**DOI:** 10.3390/s22072619

**Published:** 2022-03-29

**Authors:** Yuanhao Cui, Visa Koivunen, Xiaojun Jing

**Affiliations:** 1 School of Information and Communication Engineering, Beijing University of Posts and Telecommunications, Beijing 100876, China; cuiyuanhao@bupt.edu.cn; 2 Department of Signal Processing and Acoustics, Aalto University, 00076 Espoo, Finland; visa.koivunen@aalto.fi

**Keywords:** communication-radar coexistence, spectrum sharing, integrated sensing and communications

## Abstract

The coexistence of radar and communication systems is necessary to facilitate new wireless systems and services due to the shortage of the useful radio spectrum. Moreover, changes in spectrum regulation will be introduced in which the spectrum is allocated in larger chunks and different radio systems need to share the spectrum. For example, 5G NR, LTE and Wi-Fi systems have to share the spectrum with S-band radars. Managing interference is a key task in coexistence scenarios. Cognitive radio and radar technologies facilitate using the spectrum in a flexible manner and sharing channel awareness between the two subsystems. In this paper, we propose a nullspace-based joint precoder–decoder design for coexisting multicarrier radar and multiuser multicarrier communication systems. The maximizing signal interference noise ratio (max-SINR) criterion and interference alignment (IA) constraints are employed in finding the precoder and decoder. By taking advantage of IA theory, a maximum degree of freedom upper bound for the K+1-radar-communication-user interference channel can be achieved. Our simulation studies demonstrate that interference can be practically fully canceled in both communication and radar systems. This leads to improved detection performance in radar and a higher rate in communication subsystems. A significant performance gain over a nullspace-based precoder-only design is also obtained.

## 1. Introduction

### 1.1. Background and Motivation

The tremendous growth in new wireless systems and services has caused a shortage in the useful radio spectrum [1]. For instance, the Federal Communications Commission (FCC) and the National Telecommunications and Information Administration (NTIA) proposed adjusting the application of 150 megahertz from surveillance and air defense to radar and communication systems in the 3.5 GHz band [2,3,4,5]. Agile spectrum use and spectrum sharing among different radio systems provide promising technologies for alleviating the lack of useful spectrum.

By exploring radar spatial degrees of freedom (DoFs), the joint transmit design of a multiple-input multiple-output matrix completion (MIMO-MC) radar and a point-to-point multiple-input multiple-output (MIMO) communication system [6,7] has received considerable attention. However, the impact of multiuser communication systems is understudied. In terms of spectrum sharing in the 3.5 GHz spectrum and hardware similarity, a multicarrier radar waveform is considered to be one of the best choices for spectrum sharing between radar and the communication system. Indeed, a multicarrier signal has already been widely accepted as a physical layer modulation solution both in communication systems [8,9,10] and in the radar field [11,12,13,14].

Furthermore, a communication signal that is scattered off the target is unitized to capture mutual-information-based radar waveform designs in [15,16]. Currently, the interference signal can be projected into the nullspace of the channel matrix to avoid interference. The majority of recent projection-based radar-communication coexistence techniques, e.g., [17,18,19], project either radar or communication signals into the other system’s nullspace. Indeed, in the studies of precoder-only projection-based design [17,18,19], a multiuser communication system is simplified to a single communication user with combined signal spaces of all communication users, ignoring the mutual interference.

The radar subsystem searches for a nullspace or alternative nullspace of the communication subsystem to project a signal into it. However, the feasibility of the precoder-only design depends on the channel state matrix, which is determined by the propagation and the target environment. Furthermore, comparing with precoder-only projection-based design [17,18,19], this type of precoder design allows for avoiding interference on either subsystem but not on both.

In the case that radar and communication systems are jointly designed and co-located, the sharing of channel knowledge and interference awareness can be conveniently arranged. Consequently, optimizing transmitted waveforms and receiver processing for radar and communication systems can be performed jointly. For example, precoders and decoders in radar and communication systems may be jointly designed to construct signal spaces and orthogonal interference spaces and to obtain more effective and flexible interference management.

### 1.2. Contributions of This Work

In this paper, precoders and decoders are designed, optimized and adapted jointly using interference alignment (IA) to manage interference in coexisting multicarrier radar [20] and multiuser-multicarrier communication systems. DoF exploitation and mutual interference between radar and communication systems are studied for the coexistence of radar and multiple communication users.

The main contributions of this paper are as follows:

- The multicarrier model of [20] is extended to a more general setting in which multicarrier radar and communication systems coexist. Compared with [20], the resulting generalized multicarrier radar-communication signal model is able to capture a multicarrier radar-communication coexistence scenario and the differences between multicarrier radar and communication waveforms.
- A joint precoder–decoder design is proposed using the max-SINR criterion and IA theory for a multicarrier-multiuser radar-communication coexistence scenario. Specifically, at each user, the signal space and interference space are spanned by columns of the decoder. Consequently, mutual interference between radar and multiuser communication systems can be almost completely eliminated by the proposed joint design.
- For *K* communication users and one radar user interference channel and assuming that the IA constraint is feasible, the proposed joint precoder–decoder design is able to achieve $N_{sc}\left(K+1\right)/2$ total DoFs, which is known as the achievable DoF upper bound for the (K+1)-user interference channel with $N_{sc}$ subcarriers [21]. In other words, if radar waveforms and communication codebooks are appropriately designed, this proposed IA-based design is able to achieve the optimal total information throughput for the entire radar-communication coexistence system. Radar users could obtain better detection performance, diversity gain and interference-free DoFs compared to a subspace-based precoder-only design.

### 1.3. Brief Overview of Related Work

Various contributions have been presented in the radar and communication spectrum sharing literature. In general, these works can be classified into three main classes [22,23,24,25]: codesign, cooperation and coexistence. This paper belongs to the third category.

- **Co-design**: When hardware modification is possible, radar and communication systems can be jointly designed to maximize joint performance [4,26,27]. One example is a joint Orthogonal Frequency Division Multiplexing (OFDM) radar-communication system: information transmission and target localization tasks can be irrelevantly and simultaneously accomplished by a jointly designed system based on an OFDM signal [28,29]. Furthermore, the works in [30,31,32,33] show that communication information could be embedded into the sidelobe of the radar waveform to develop a co-designed dual-function system.
- **Cooperation**: Limited information can be shared between radar and communication systems to effectively mitigate interference rather than isolate systems. Bliss et al. presented cooperative joint radar-communications inner bounds in [34,35,36] and extended this concept to MIMO systems [37]. Radar waveforms can be embedded as a pilot signal of communication systems in a doubly selective channel for detection and channel state estimation [38].
- **Coexistence**: When radar and communication systems coexist, interference management is the key issue. Practically, if the interfering energy is weak or the signal structure is unknown, an interfering signal may be treated as interference, e.g., interference from a Wi-Fi transmitter to a radar receiver. Furthermore, physical separation was introduced in [39,40] to reduce the interfering energy below the noise level. Most of the prior radar-communication spectrum sharing approaches address interference management by exploiting orthogonality [17,18,19,41,42,43] or designing radar and communication signals while guaranteeing acceptable performance [6,7,44,45]. A robust precoder that minimizes power is proposed for coexistence between MIMO radar and downlink multiuser MIMO communication systems in [46]. The works in [22,35] explore information-theoretic bounds for a single joint radar-communication user. The theoretical foundation of joint radar-communication research is established in these studies.

However, the channel capacity of a multiuser radar-communication interference channel [47,48,49] is still an open problem. An inspiring study [50] showed that the IA scheme could achieve the total DoF upper bound as $\frac{K}{2}$ for a *K*-user interference channel. The key idea of IA is a linear precoding and decoding technique to design the signal space and orthogonal interference space for each user. Essentially, the IA is a trade-off between DoFs and interference in a multiuser interference channel.

The definition of DoF is clear in the communication literature but is rarely studied in the radar literature. In general, radar extracts information from targets [51]. The number of frequency-domain DoFs of radar equals the rank of the target response matrix, implying the capability of resolving targets [7]. Maximizing the DoF upper bound corresponds to maximizing the achievable capability of resolving targets. When the independent random variables follow an exponential distribution and the Neyman–Pearson detection strategy is applied, maximizing the frequency DoFs corresponds to maximizing the diversity gain because the diversity gain for each independent variable in the exponential family is 1 [52].

Although there is a large amount of results in the radar-communication coexistence category, the problem of interference management has not been thoroughly studied in multiuser communication scenarios considering mutual interference and DoF exploitation simultaneously.

**Notation**: In this paper, matrices are denoted by capital letters, vectors are denoted by boldface, Re{·} means the real part of a complex signal, ∘ is the Hadamard product, $A_{\left[i\right]}$ means matrix A for the *i*th transmitter and receiver, $A_{\left[ij\right]}$ means matrix A with respect to the *i*th radio transmitter and *j*th radio receiver, $A_{\left[R\right]}$ means matrix A with respect to the radar only, $A_{N\times M}$ denotes an N×M-dimensional matrix, ⌊·⌋ is the floor function, $A^{H}$ is the Hermitian transpose of matrix A, and $\bar{A}$ is matrix A on the reciprocal interference channel. Moreover, the math symbols are summarized in Abbreviations.

## 2. Generalized Multicarrier Radar-Communication Coexistence Model

In this section, we extend a generalized multicarrier radar signal model [20] to more general settings, where radar and communication systems coexist and share the same radio spectrum. We start from a single-input single-output (SISO) point-to-point generalized multicarrier signal model to capture multicarrier radar and multicarrier communication signals. Then, the signal model is applied for K+1 radar and communication users, where a radar user coexists with *K* communication users.

### 2.1. Transmitter

The discrete-time signal model in Appendix A can be rewritten in a compact matrix form,

(1) $$Y_{T}=\left(\Omega \circ B\right)PCS$$

where ∘ is the Hadamard product. $Y_{T}$ is an $N_{sc}\times M$-dimensional sample matrix that occupies *M* time slots and $N_{sc}$ subcarriers for $k=\{0,1,2,\cdots ,MN_{sc}-1\}$ samples, denoted as a resource block for communication or pulse for radar. The matrix $P\in C^{N_{sc}\times N}$ denotes the linear frequency precoding matrix that contains subcarrier weights. Matrix $C\in C^{N\times N_{p}}$ represents a coding matrix, where $N_{p}$ denotes the length of the uncoded data sequence. Matrix $S\in C^{N_{p}\times M}$ contains transmitted data S=[s(1)⋯s(M)], where s(M) is an $N_{p}$ data vector for *M* time slots.

**Figure 1:**
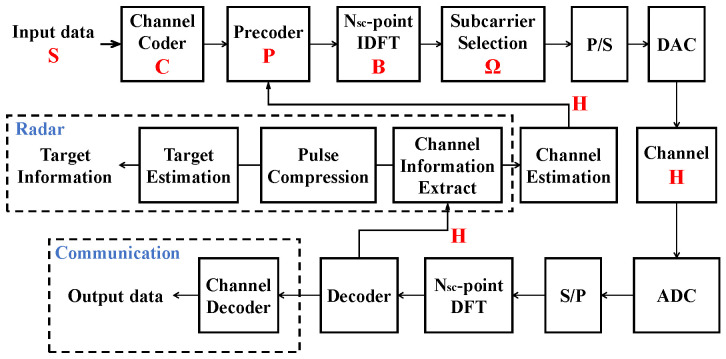
Multicarrier radio system for both multicarrier communication and multicarrier radar systems or a colocated radar-communication system. In the case of a radar or communication user, the dashed boxes of communication or radar in the block diagram are ignored. P/S (S/P) denotes parallel-to-serial or serial-to-parallel conversion, and DAC/ADC represents a digital-to-analog converter or analog-to-digital converter.

A block diagram of a multicarrier system for radar and communication coexistence is shown in Figure 1. This figure illustrates how the generalized multicarrier radar-communication signal model can be applied to generate signals at the transmitter and process them at the receiver. The input data will be processed by the channel coder, precoder, IDFT, subcarrier selection, parallel-to-serial conversion and digital-to-analog converter to generate a multicarrier radar communication signal. After decoding, the channel information is estimated in the communication subsystem, or target information is estimated in the radar subsystem.

The matrix Ω is an $N_{sc}\times N_{sc}$-dimensional selection matrix, in which the elements of Ω are either 1 or 0 to indicate whether subcarriers in matrix $Y_{T}$ are active and to manage the pilot symbol. For example, a widely used radar pulse and communication signal can be controlled by deactivating resource block elements of Ω. In practice, the selection matrix selects carriers to sharpen the waveform patterns, various waveforms can be generated by enabling or disabling its elements, such as a step approximation of the up-chirp pulse, a pseudo-random frequency-hopping pulse and a multicarrier communication signal with a Comb-type pilot subcarrier.

Matrix $B\in C^{N_{sc}\times N_{sc}}$ in (1) is considered to be a modulation matrix. In an OFDM system, matrix B is a Vandermonde matrix, and its elements are associated with subcarriers as follows [20]:

(2) $$B=\frac{1}{\sqrt{N_{sc}}}\left(\begin{array}{cccc} 1 & 1 & \cdots & 1\\ 1 & \beta & \cdots & \beta^{N_{sc}-1}\\ 1 & \beta^{2} & \cdots & \beta^{2(N_{sc}-1)}\\ \cdots & \cdots & \cdots & \cdots \\ 1 & \beta^{N_{sc}-1} & \cdots & \beta^{\left(N_{sc}-1\right)\left(N_{sc}-1\right)} \end{array}\right)$$

where

(3) $$\beta =e^{j2\pi \Delta f(\frac{T_{c}}{N_{sc}})},\;\mathrm{for}\;\mathrm{both}\;\mathrm{radar}\;\mathrm{and}\;\mathrm{communication}$$

denotes baseband subcarriers without a carrier/sampling index. $T_{c}$ is the symbol duration of the multicarrier system, and Δf is the subcarrier spacing. The time duration between row elements is (*k* mod $N_{sc}$) sample periods. The same active/inactive subcarrier pattern is applied for the time duration of each radar subpulse/communication symbol. This pattern justifies the (*k* mod $N_{sc}$) time duration above. In the special case of an OFDM signal, matrix B is an inverse discrete Fourier transform (IDFT) matrix, $B^{H}B=I_{N_{sc}\times N_{sc}}$, where $T_{c}=\frac{1}{\Delta f}$.

In a multicarrier communication system, the precoder functions as a multimode beamformer that allocates signal power according to channel quality, similar to the MIMO spatial precoder in [53]. Furthermore, the power leakage could be optimized by an orthogonal precoding design [54]. In a multicarrier radar, P is applied as a multimode beamformer, projecting the radar signal into a designed subspace. Moreover, the data channel coding rate is $\frac{N_{p}}{N}$ when coded data are transmitted in parallel and $N-N_{p}$ is the coding redundancy [55].

The code rate upper bound of coding matrix C is shown in [56]. As channel coding increases the channel reliability by introducing redundancy, it could increase the number of columns in the data matrix, i.e., $\frac{N_{p}}{N}\leq 1$. Matrix C facilitates pulse compression and consequently improves the radar resolution while functioning as a channel coder to improve the reliability of communication transmission [20]. In this paper, we consider the IA criterion to cancel the interference of both systems.

As the transmitted waveform is always known by a radar receiver, without a loss of generality, we assume that the radar waveform is an all-ones matrix. Then, $SS^{H}=M1_{N_{p}\times N_{p}}$ for the radar signal. The transmitted payload data in the communication system is unknown to the receiver. It may be modeled with a complex Gaussian distribution, while its power is considered to be $E\left(SS^{H}\right)=\sigma_{S}^{2}I_{N_{p}\times N_{p}}$.

### 2.2. Receiver

Both the multicarrier radar and communication receiver in the coexistence system will obtain a radar response from the target reflection or an observation from an active transmitter in a communication system. For a radar receiver, assume that a single moving target is located at direction θ. By ignoring interference, a general form of the received signals before decoding may be written as follows:

(4) $$Y_{R}=H\left(\theta \right)\left(\Omega \circ I_{N_{sc}\times N_{sc}}\right)PCS+W,$$

where matrix $Y_{R}$ is an $N_{sc}\times M$-dimensional received signal matrix. W denotes the $N_{sc}\times M$-dimensional complex white Gaussian noise at the receiver with covariance matrix, $E\left(WW^{H}\right)=\sigma_{W}^{2}I_{N_{sc}\times N_{sc}}$. A detailed derivation of the model is presented in Appendix B.

The channel frequency response matrix denoted by $H\left(\theta \right)\in C^{N_{sc}\times N_{sc}}$ could be estimated for each *L* symbol duration under the block fading channel assumption. When an OFDM signal is employed, the channel state matrix is a diagonal matrix $H\left(\theta \right)=\mathrm{diag}\{h_{1}\left(\theta \right),h_{2}\left(\theta \right)\cdots ,h_{N_{sc}}\left(\theta \right)\}$, where $h_{1}\left(\theta \right)$ is the channel frequency response at the first subcarrier.

Considering the radar task at hand, the channel state matrix describes the combined effects of target response, scattering, channel fading, Doppler shift, and power decay with distance. Among these parameters, the direction of arrival (DoA), Doppler shift, and target response are of particular interest for the radar system. Note that if multiantenna transceivers are used, spatial processing, such as DoA estimation may be performed. If a single-antenna system is employed, the DoAs can be estimated using a mechanically rotating directional antenna, as in many classical radar systems [57]. The communication receiver treats Doppler shifts, multipath effects and power decay as channel distortion. To ensure reliability of data transmission, channel coding and frequency precoding are typically employed. One needs to design a channel coding matrix C and a precoding matrix P to address the dynamic nature of H(θ).

**Figure 2:**
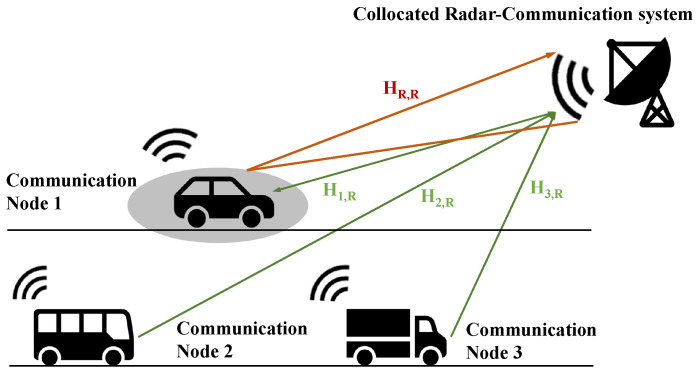
A practical vehicle networking example, in which the colocated radar-communication system coexists with a multiuser communication network.

In particular, for a colocated radar-communication node as shown in Figure 2, the communication subsystem could easily share a channel state matrix with a radar subsystem. Moreover, if the target also carries a cooperating communication system, the performances of the channel and target estimations may both benefit from a joint estimating procedure. A practical example of such a system is a vehicle radar and vehicle-to-vehicle communication network.

### 2.3. Multiuser Radar-Communication Spectrum Sharing Scenario

In this subsection, we consider a simplified K+1 user radar-communication spectrum sharing scenario, which consists of *K* SISO communication users and a SISO multicarrier monostatic radar user. They share $N_{sc}$ subcarriers and the same frequency band.

Consequently, the sum of the numbers of possible transmitter and receiver (TX-RX) pairs (including the signal channel and interference channel) is K(K+1). Each user is considered to be an intended TX-RX pair (matched link) transmitting a useful signal. Hence, the sum of useful signal channels is *K*. Interference occurred in unintended TX-RX pairs (mismatched link), which may be from radar TX to one communication RX, from one communication TX to radar RX or from communication TX to another unintended communication RX. The interference channels correspond to $K^{2}$ unintended links.

A monostatic radar is colocated with one of these communication nodes. The joint radar communication system is considered to be a radar and a communication user separately, as illustrated in Figure 2.

In this network configuration, we denote by A the set of communication users and by B the set of radar users, where |A|=K,|B|=1. Here, |A| denotes the cardinality of set A. The colocated multicarrier monostatic radar illuminates one target in the direction θ. Note that radar is likely forming narrow beams or has a highly directional antenna such that it can only interfere with part of the communication nodes.

Recall that the signals are experiencing a block fading channel. Before decoding, the received multicarrier signal at the *i*th receiver in the K+1-user radar-communication coexistence scenario may be written as follows:

(5) $$\begin{array}{cc} Y_{R} & =\underset{\mathrm{Radar}\;\mathrm{Signal}}{\underbrace{H_{\left[R\right]}\left(\theta \right)\left(\Omega_{\left[R\right]}\circ I_{N_{sc}\times N_{sc}}\right)P_{\left[R\right]}C_{\left[R\right]}S_{\left[R\right]}}}\\ \; & +\underset{\mathrm{Communication}\;\mathrm{Signal}}{\underbrace{\sum_{j\in A}H_{\left[ij\right]}\left(\Omega_{\left[j\right]}\circ I_{N_{sc}\times N_{sc}}\right)P_{\left[j\right]}C_{\left[j\right]}S_{\left[j\right]}}}\\ \; & +W_{\left[i\right]} \end{array}$$

where $Y_{R[i]}$ is the received signal for the *i*th user. Here, the *i*th receiver may refer to all *K* communication receivers and radar receiver i∈A∪B. $\Omega_{\left[R\right]}$ denotes the selection matrix associated with adaptive waveform design, e.g., pseudo-random pulse or multi-pulse radar. Due to the block fading channel assumption, the channel coherence interval is *L* pulses/resource blocks. Channel coding matrix C will remain unchanged because it is designed based on H in both communication and radar subsystems. Consequently, $\Omega_{\left[R\right]}$, $\Omega_{\left[j\right]}$, P and $S_{\left[j\right]}$ may vary over *L* pulses/resource blocks.

To describe the mode in detail, some key conditions are stated as follows:

- Channel State Information (CSI): The channels $H_{\left[ii\right]}$ and $H_{\left[ij\right]}$ and the target response matrix ${H(\theta )}_{\left[ii\right]}$ are considered to be perfectly estimated at the communication and radar transmitters, respectively. CSI estimation has been applied in communication systems for a long time. For radar, one feasible method is to treat a known radar waveform as a shared pilot in both radar and communication systems. The pilot-aided approach can estimate all the channel information between radar and communication users [38]. Another approach is to embed the same pilot signal in both the radar coherence interval and communication frames [7]. The benefit of the distributed IA method is that CSI is available locally. Moreover, channel reciprocity may be exploited for interference channels.
- Synchronization: Both radar and communication systems are assumed to be synchronized. If they are colocated, then they may share the same clock, in which case, synchronization is not an issue. The other subsystems need to be synchronized in a similar manner to any multiuser communication system; the clock synchronization may be easier in communication systems but still feasible for radar. Existing radar clock synchronization technology may be employed, such as using a Global Navigation Satellite System (GNSS) [58,59], using a pilot signal [60] or using an OFDM frame [61] to achieve time and frequency synchronization [28].
- Shared Information: As shown above, the following information is shared among all users: selection matrix $\Omega^{\left(l\right)}$ and data covariance $\sigma_{S}$. This shared information is needed to calculate the transmitted signal power and consequently to solve the following optimization problem at the *l*th time slot. Alternatively, the transmitted signal power can be constrained by the power limitation.
- Doppler: In this paper, radar is detecting a far-field point target, and all communication channels are experiencing block fading. The Doppler shift is assumed to be constant during a coherent interval for the *L* pulse.
- Schedule: The feedback of the channel state matrix, transmission of clock synchronization and shared information call for schedules between the communication and radar systems. Providing channel feedback is common in most modern communication systems and part of the standards. A radar system can also take advantage of feedback and estimate channels. One feasible approach is to transmit information across the radar-coherent interval based on a time division multiplex [7].

## 3. Max-SINR Joint Precoder–Decoder Design

IA is an emerging DoF-based interference management technique in wireless communications that aligns the interference caused by other users in an interference signal subspace that is orthogonal to the user-desired signal subspace [62]. This technique can be applied in the time, frequency or spatial domain. Furthermore, in a high-SNR regime, this technique can achieve high-interference elimination and offer a $\frac{K}{2}$ achievable total DoF upper bound in the *K*-interference-communication-user scenario without frequency/time/spatial symbol extension [21,50]. Symbol extension means additional diversity, for example, by expanding bandwidth or adding antennas in this paper. At the same time, the traditional orthogonal spectrum allocation, which allocates a nonoverlapping signal to each user, could only obtain $\frac{1}{K}$ interference-free DoFs.

In this section, we propose a joint precoder–decoder design using the max-SINR criterion and IA approach to solve the radar-communication coexistence problem. Communication receivers are interfered with by radar via a direct path. As the radar signal reflected from the target may also have a high power, we could treat the scattered radar signal as noise for the communication receiver and consequently a medium SNR scenario. IA in a high-SNR regime typically focuses on minimizing the leakage interference while ignoring noise. However, in this radar-communication coexistence problem in a medium-SNR regime, it is necessary to take the noise into account. Hence, we will employ the max-SINR criterion for our design.

### 3.1. Ideal IA Constraints

The ideal IA constraints in a multicarrier radar-communication coexistence scenario can be written as follows [62]:

(6a) $$\begin{array}{c} Q_{\left[i\right]}^{H}H_{\left[ij\right]}P_{\left[j\right]}=0_{d_{\left[i\right]}\times d_{\left[j\right]}}, \end{array}$$

(6b) $$\begin{array}{c} \mathrm{rank}\left(Q_{\left[i\right]}^{H}H_{\left[ii\right]}P_{\left[i\right]}\right)=d_{\left[i\right]}, \end{array}$$

(6c) $$\begin{array}{c} \forall i\neq j;\forall i,j\in A\cup B, \end{array}$$

where $0_{d_{\left[i\right]}\times d_{\left[j\right]}}$ is a $d_{\left[i\right]}\times d_{\left[j\right]}$ zero matrix, $Q_{\left[i\right]}$ is an $N_{sc}\times N_{\left[i\right]}$-dimensional decoder matrix of the *i*th communication node, and recall that $N_{\left[i\right]}=d_{\left[i\right]}$.$d_{\left[i\right]}$ denotes the user-desired DoFs for the *i*th user. The channel matrix of the interference channel between the *i*th radio node and the *j*th radio node is $H_{\left[ij\right]}$, and the matrix of the signal channel at the *i*th user is $H_{\left[ii\right]}$. When i∈A, $H_{\left[ii\right]}$ is a target response matrix $H_{\left[ii\right]}\left(\theta \right)$, which is of interest to the radar subsystem.

Constraint (6a) means that the precoder is designed to project the interference from the *j*th radio transmitter to the *i*th receiver’s nullspace. The nullspace is designed by decoder $Q_{\left[i\right]}$ to align interference from all *j* transmitters, ∀i≠j;∀j∈A∪B. Equation (6b) implies that the designed signal space should satisfy the desired DoFs. The ideal IA constraints design precoders $P_{\left[j\right]}$ to project interference such that it is aligned in the nullspace of $Q_{\left[i\right]}$ and design $Q_{\left[i\right]}$ to guarantee that the signal space and interference space exits.

Constraint (6b) is automatically satisfied almost surely in the case where the channel matrix does not have a special structure [50], for example, in the MIMO case. However, (6b) must be taken into account due to the diagonal channel state matrix in the case of an OFDM waveform.

As the obtained precoder and decoder solution is not unique, there may be some solutions that project both useful signals and interference signals into the nullspace [63]. For example,

$$Q=\left[\begin{array}{c} 0\\ 1 \end{array}\right]P=\left[\begin{array}{c} 1\\ 0 \end{array}\right]$$

will project any signal into the nullspace for any diagonal channel state matrix. Intuitively, it follows from (6a), and (6b) may be described as

(7a) $$\begin{array}{c} 0=Q_{\left[i\right]}^{H}H_{\left[ij\right]}P_{\left[j\right]}, \end{array}$$

(7b) $$\begin{array}{c} {\tilde{H}}_{\left[ii\right]}=Q_{\left[i\right]}^{H}H_{\left[ii\right]}P_{\left[i\right]}, \end{array}$$

(7c) $$\begin{array}{c} \forall i\neq j;\forall i,j\in A\cup B \end{array}$$

where expression (7a) indicates that after the application of the precoder and decoder, an undesired signal from the *j*th transmitter will be projected into the nullspace that is designed by the *i*th decoder. The user-desired interference-free signal channel matrix ${\tilde{H}}_{\left[ii\right]}$ is a square rank $d_{\left[i\right]}$ matrix. Recall that the radar channel matrix includes the target information that the radar is interested in when i∈A. This equivalent channel matrix offers an intuitive way to understand how IA-based precoders and decoders process radar signals.

### 3.2. Reciprocity and Feasibility of IA

In a radio link, the roles of the receiving and transmitting antennas are functionally interchanged, while the instantaneous transfer characteristics of the radio channel remain unchanged. This channel reciprocity can be exploited in IA design. The reciprocity of IA [62] comes from the identity IA constraints between the original interference channel and the reciprocal interference channel, where the original precoder and decoder are considered to be a reciprocal decoder and precoder, respectively. In other words, IA constraints are still feasible after the signal direction is reversed. Using reciprocity, the IA constraints of (7) can be written as follows:

(8) $$0={\bar{Q}}_{\left[j\right]}^{H}{\bar{H}}_{\left[ji\right]}{\bar{P}}_{\left[i\right]},{\bar{\tilde{H}}}_{\left[ii\right]}={\bar{Q}}_{\left[i\right]}^{H}{\bar{H}}_{\left[ii\right]}{\bar{P}}_{\left[i\right]},\forall i\neq j;\forall i,j\in A\cup B$$

where $\bar{P}$ and $\bar{Q}$ denote the precoder and decoder on the reciprocal channel, ${\bar{Q}}_{\left[i\right]}=P_{\left[i\right]},{\bar{P}}_{\left[i\right]}=Q_{\left[i\right]},$∀i∈A∪B. Furthermore, $0_{d_{\left[i\right]}\times d_{\left[j\right]},\left[ij\right]}$ denotes the interference nullspace from the *i*th receiver to the *j*th transmitter on the reciprocal channel. ${\bar{\tilde{H}}}_{\left[ii\right]}$ denotes the channel matrix for the *i*th user itself on the reciprocal channel. The reciprocity of IA does not change the user-desired DoFs of each user while projecting an undesired signal into the nullspace. However, it plays an important role in the following distributed iteration algorithm.

As with the communication signal, the DoFs of a radar subsystem need to be properly chosen to realize IA. The radar subsystem also works better with more DoFs available to achieve a more flexible waveform design and better diversity gain. In this paper, we assume that the number of desired DoFs is predetermined. The DoFs of radar need to satisfy the feasibility condition of IA, which is written as follows:

(9) $$\left\{\begin{array}{cc} \; & d_{\left[R\right]}+d_{\left[i\right]}\leq N_{sc},\forall i\in A\cup B\\ \; & 2d_{\left[R\right]}\left(N_{sc}-d_{\left[R\right]}\right)-\sum_{i\in A_{sub}}d_{\left[R\right]}d_{\left[i\right]}\geq 0,\forall A_{sub}\subset A\cup B. \end{array}\right.$$

This condition can easily be formulated based on Theorem 2 in [64]. Note that (9) is a necessary condition. IA requires finding feasible signal strategies while the channel matrix is fixed. However, the feasibility is established in a reverse way, where the strategy is fixed and the study channel matrix is set for which strategy is feasible. The space of strategies can be represented by the product of Grassmannians (7, [64]). If IA is feasible, then the dimension of projection between the strategy space and channel matrix must be non-negative [64]. Furthermore, if $d_{\left[i\right]}=d_{\left[R\right]}=d$, i.e., the desired DoFs of the communication and radar nodes are identical and equal to *d*, the IA is feasible if and only if

(10) $$d\leq \frac{2N_{sc}}{K+1}.$$

This condition degenerates into a necessary condition when $d_{\left[i\right]}=d_{\left[R\right]}=d=1,K\geq 3$, where (Theorem 1, [65])

(11) $$K\leq 2N_{sc}-2.$$

The general feasibility condition of a proper IA network with multiple streams for each user remains an open problem.

### 3.3. Distributed Max-SINR Precoder–Decoder Design

Based on IA theory, (6a)–(6c) could then be formulated as an interference minimization problem as follows:

(12) $$\begin{array}{ll} \min_{P_{\left[i\right]},Q_{\left[i\right]}} & Q_{\left[i\right]}^{H}H_{\left[ij\right]}P_{\left[j\right]}\\ s.t.\;\; & \mathrm{rank}\left(Q_{\left[i\right]}^{H}H_{\left[ii\right]}P_{\left[i\right]}\right)=d_{\left[i\right]}. \end{array}$$

However, the interference minimization criteria are not optimal when taking noise and the radar channel matrix into account. Without loss of generality, (6a)–(6c) could be formulated as a SINR optimization problem as follows:

(13a) $$\begin{array}{ll} \max_{P_{\left[i\right]},Q_{\left[i\right]}} & \frac{Q_{\left[i\right]}^{H}H_{\left[ii\right]}A_{\left[i\right]}A_{\left[i\right]}^{H}H_{\left[ii\right]}^{H}Q_{\left[i\right]}}{Q_{\left[i\right]}^{H}\left(\sum_{j=1}^{Z_{\left[i\right]}}H_{\left[ij\right]}A_{\left[j\right]}A_{\left[j\right]}^{H}H_{\left[ij\right]}^{H}+W_{\left[i\right]}W_{\left[i\right]}^{H}\right)Q_{\left[i\right]}} \end{array}$$

(13b) $$\begin{array}{ll} s.t.\; & \mathrm{rank}\left(Q_{\left[i\right]}^{H}H_{\left[ii\right]}P_{\left[i\right]}\right)=d_{\left[i\right]}. \end{array}$$

Here, noise power is described by $E\left(W_{\left[i\right]}W_{\left[i\right]}^{H}\right)=\sigma_{W}^{2}I_{N_{sc}\times N_{sc}}$, and $Z_{\left[i\right]}$ is the number of interfering sources for the *i*th receiver. Projecting interference into the designed nullspace with fixed SNR implies maximizing the SINR as (13a).

Let us recall (1); the signal power emitted from the *i*th transmitter may be written as

(14) $$A_{\left[j\right]}A_{\left[j\right]}^{(l)H}=\left\{\begin{array}{c} M\left(\Omega_{\left[R\right]}\circ I_{N_{sc}\times N_{sc}}\right)P_{\left[R\right]}Tr(C_{\left[R\right]}1\\ C_{\left[R\right]}^{H})P_{\left[R\right]}^{H}{\left(\Omega_{\left[R\right]}\circ I_{N_{sc}\times N_{sc}}\right)}^{H}\;\mathrm{if}\;j\in B\\ \sigma_{S}^{2}\left(\Omega_{\left[j\right]}\circ I_{N_{sc}\times N_{sc}}\right)P_{\left[j\right]}Tr\left(C_{\left[j\right]}C_{\left[j\right]}^{H}\right)\\ P_{\left[j\right]}^{H}{\left(\Omega_{\left[j\right]}\circ I_{N_{sc}\times N_{sc}}\right)}^{H}\\ \mathrm{if}\;j\in A. \end{array}\right.$$

Recall that the set A contains communication users. Some of these communication receivers are interfered by radar, denoted as the subset $A_{r}$, and the complement set of communication users not interfered by radar is denoted as $A_{c}$. If the *i*th communication receiver is interfered by radar, $i\in A_{r}$, it will experience interference from $Z_{\left[i\right]}=K-1$ communication transmitters and one radar transmitter, ∀j∈A∪B,i≠j. If this receiver is not interfered by radar, $i\in A_{c}$, its interference is caused by $Z_{\left[i\right]}=K-1$ communication transmitters only, ∀j∈A,i≠j. If this receiver is a radar receiver, then it is subject to interference from all $Z_{\left[i\right]}=K$ communication transmitters, ∀j∈A.

Here, we use the commutative law of the Hadamard product and the generalized radar-communication coexistence signal model in Section II-C. The trace term in (14) corresponds to the signal power before the precoding and modulation operation.

The objective function is maximized in an iterative manner. In each iteration, we maximize the objective function (13a) to find $Q_{\left[i\right]}$ at each receiver. Then, at each transmitter, we will find the original $P_{\left[i\right]}$ according to (13a). At this time, (13a) can be further simplified to accelerate the calculations.

The interference plus noise covariance matrix for the *i*th receiver may be written as follows:

(15) $$D_{\left[i\right]}=\sum_{j=1}^{Z_{\left[i\right]}}H_{\left[ij\right]}A_{\left[j\right]}A_{\left[j\right]}^{H}H_{\left[ij\right]}^{H}+W_{\left[i\right]}W_{\left[i\right]}^{H}.$$

Recall that all terms except $Q_{\left[i\right]}$ are fixed to find $P_{\left[i\right]}$. When maximizing (13), $Q_{\left[i\right]}$ is normalized to limit the decoder element scope for eigenvectors and have easier calculations. It may be given by the following:

(16) $$Q_{\left[i\right]}=\frac{V_{d_{\left[i\right]}}\left(D_{\left[i\right]}^{-1}H_{\left[ii\right]}P_{\left[i\right]}P_{\left[i\right]}^{H}H_{\left[ii\right]}^{H}\right)}{∥V_{d_{\left[i\right]}}\left(D_{\left[i\right]}^{-1}H_{\left[ii\right]}P_{\left[i\right]}P_{\left[i\right]}^{H}H_{\left[ii\right]}^{H}\right)∥},$$

where $V_{d_{\left[i\right]}}\left(A\right)$ denotes the eigenvectors corresponding to the $d_{\left[i\right]}$ smallest eigenvalues of A.

The objective function in (13) is not convex. However, we could find a solution by employing channel reciprocity and a distributed iterative algorithm. This algorithm is guaranteed to converge, but it may not necessarily find the global optimum [62]. By taking all radio nodes into account, we can write an algorithm for solving this optimization problem in (13). See Algorithm 1 for detailed steps. The reciprocal interference channel is still IA feasible by choosing the original precoder as a decoder of the reciprocal interference channel and the original decoder as a precoder, respectively. The algorithm finds $Q_{\left[i\right]}$ and $P_{\left[i\right]}$ iteratively in two stages.
**Algorithm 1** Max-SINR design algorithm1:Estimate radar channel $H_{\left[R\right]}$ and radar’s interference channel $H_{\left[iR\right]}$2:Initialize $P_{\left[i\right]}$,$Q_{\left[i\right]}$ with independent row vectors, ∀i∈A∪B3:**repeat**4:   **repeat**5:     Identify location and type of *i*th node, ∀i∈A∪B6:     Choose $Z_{\left[i\right]}$ according to its location and type7:     Calculate transmitted signal power according to Equation (14)8:     Calculate interference pulse noise covariance matrix $D_{\left[i\right]}$ according to Equation (15)9:     Find $N_{sc}\times d_{\left[i\right]}$ matrix $Q_{\left[i\right]}$ on each receiver according to (16)10:   **until** All $Q_{\left[i\right]}$ are found11:   **repeat**12:     Use channel reciprocity13:     Identify location and type of *i*th node, ∀i∈A∪B14:     Choose $Z_{\left[i\right]}$ according to its location and type15:     Calculate power of reciprocal transmitted signal according to Equation (14)16:     Calculate interference pulse noise covariance matrix $D_{\left[i\right]}$ of reciprocal signal according to Equation (15)17:     Calculate $N_{sc}\times d_{\left[i\right]}$ matrix $P_{\left[i\right]}$ on each reciprocal receiver according to (16)18:   **until** All $P_{\left[i\right]}$ are calculated19:   Check rank of $Q_{\left[i\right]}^{H}H_{\left[ii\right]}P_{\left[i\right]}$ to verify (14b).20:**until** (14b) is satisfied and (13) has converged or iteration count is larger than tolerance threshold

The proposed distributed algorithm operates for finding precoders and decoders in an alternating manner. We start by finding solutions for $Q_{\left[i\right]}$ at each receiver from (13), with fixed $P_{\left[i\right]}$, $P_{\left[j\right]}$, $Q_{\left[j\right]}$,∀i≠j;∀i,j∈A∪B. Precoders $P_{\left[i\right]}$ are found using fixed decoders $Q_{\left[i\right]}$, $Q_{\left[j\right]}$, $P_{\left[j\right]}$, ∀i≠j;∀i∈A∪B, where *j* is selected based on its node type and location. In the next stage, we will reverse the signal direction, the original transmitter will be treated as a receiver, and the original receiver will be treated as a transmitter.

Consequently, a solution of decoders can be found, actually the original $P_{\left[i\right]}$’s solution for each *i*th receiver (original transmitter) from (13), while fixing $Q_{\left[i\right]}$, $Q_{\left[j\right]}$ and $P_{\left[j\right]}$, where *j* is chosen based on its node type and location. For each precoder and decoder, the rank of signal space is checked after one solution is obtained. This iteration will continue until convergence, which is evaluated by comparing (13a) to a threshold value or until the iteration count reaches its maximum value.

The constraint (13b) may also be relaxed by considering matrix dimension *N* as $d_{\left[i\right]}$ at the *i*th user. This condition guarantees that a trivial and useless solution of all zeros is avoided. Consequently, we design an $N_{sc}\times d_{\left[i\right]}$ precoder and a $d_{\left[i\right]}\times N_{sc}$ decoder for the *i*th node.

Note that the precoder and the decoder for both radar and communication subsystems in a colocated node could be found jointly by the proposed algorithm. The difference is that the precoder of radar and communication subsystems in a colocated node will be designed simultaneously at the first stage and the decoder at the second stage, while precoders and decoders of other nodes are designed in each node in a distributed manner. In cases where radar and communication subsystems are colocated and consequently suffer from the same interference, the interference channel matrix that is estimated by the communication system could easily be used in radar precoder and decoder design. Furthermore, if noise statistics are the same in both the radar and communication subsystems in the colocated node, the interference plus noise covariance matrix is also identical. It could be shared between the subsystems to reduce the calculation time, by using the same memory or optical fibers.

## 4. Simulation Examples

In this section, we present simulation results to demonstrate the performance of the proposed max-SINR joint precoder–decoder design algorithm. The simulation is done using MATLAB 2019b and a desktop with an i7-10700k processor. We consider a four-user interference channel where three communication users and one radar user colocated with one of the communication users. Each node employs a multicarrier signal model with $N_{sc}=8$ subcarriers. Assume that communication users desire one DoF and radar desires three DoFs, which is the highest practical achievable DoFs for the radar subsystem under (9). We also assume that zero mean Gaussian noise with variance $\sigma_{W}^{2}=1$ is present at each receiver. The power of the payload signal is $\sigma_{S}^{2}=1$ for each communication user.

Consider that coding matrix $C_{\left[i\right]}C_{\left[i\right]}^{H}=I$ is an identity matrix. The far-field point target is at azimuth angle $\theta =0^{\circ }$. The total number of samples is 500. The initial estimates of the precoder and decoder are obtained from independent row vectors. One of these three communication users is not interfered with by radar. Increasing the transmit power at each transmitter will also increase the interference observed by the other receivers. The channel matrices are generated randomly according to the block fading channel.

We evaluate the system performance by the sum of all the SINRs in the coexistence scenario. The performances of the communication and radar subsystems clearly depend heavily on the SINR values at the receivers. Radar performance in the detection task is studied using a ROC curve, which evaluates the performance of a radar detector by plotting the probability of detection (Pd) versus the probability of false alarm (Pfa) for a given conditions.

We compare the performance of the proposed method with the switched small singular value space projection (SSSVSP) in [18]. SSSVSP is an extension of nullspace-based precoder design in which the nullspace has been expanded to include the subspace spanned by singular vectors. These singular vectors correspond to small singular values that are selected based on a threshold value. However, it still faces the drawback that it cannot handle mutual interference between radar and communication systems. In the following simulations, we compare the proposed algorithm with SSSVSP when interference from radar is projected into communication users’ switched small singular value space.

**Figure 3:**
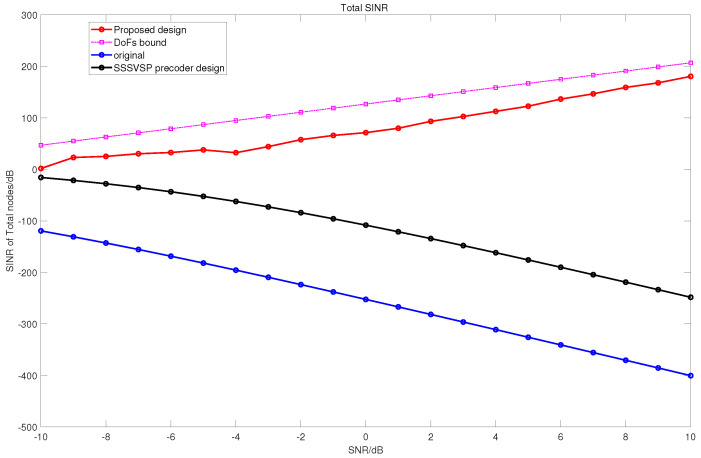
The total SINR, which is the sum of SINR values at all receivers for the proposed design, DoF bound, SSSVSP, and original signal for coexistence between the radar and communication network.

In Figure 3, we compare the total SINR values from all radio receivers, including communication and radar.

For fairness in the comparison of DoFs, the number of antennas in SSSVSP is selected to be eight. Indeed, the multicarrier signal can be mathematically regarded as a special case of a MIMO system in which the channel matrix is diagonal. For the proposed method, the SINR increases as a function of SNR, whereas the other approaches experience more interference, and consequently their SINR values decrease. However, the SSSVSP and original signal (without precoder or decoder) method decrease with it.

This result may be due to the projection of the radar signal to the nullspace of all communication receivers while ignoring the interference experienced at the radar receiver and interferences among the communication nodes. Moreover, if the signal powers increase at the transmitters, it will increase the interference power at the other receivers. The proposed method approaches the DoF upper bound as the SNR increases. The SINR of the proposed design increases as a function of SNR because interferences are almost completely eliminated. We also observe a good total SINR improvement in a medium-SNR regime.

**Figure 4:**
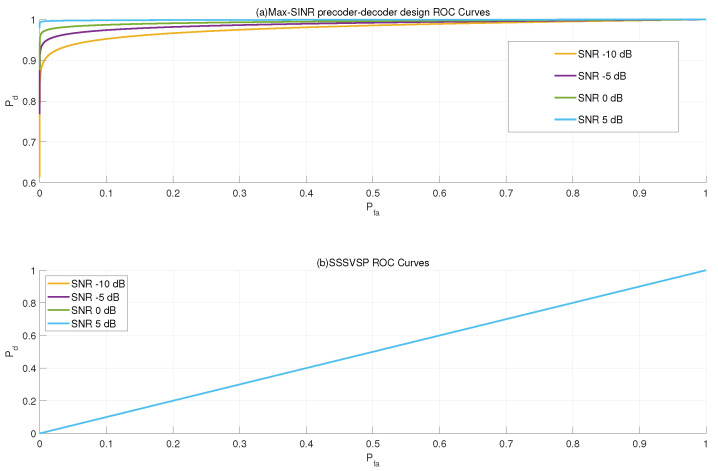
The radar’s ROC curve comparison between the proposed max-SINR joint precoder–decoder design (**a**) and SSSVSP (**b**) in −10, −5, 0 and 5 dB SNR.

In Figure 4, Neyman–Pearson detectors with different false alarm constraints are compared to the proposed design and SSSVSP method using ROC curves of the radar subsystem. Different SNR levels are considered. All radio transmitters are assumed to be active such that every receiver suffers from interference from all other transmitters. The interference experienced by different receivers will increase with signal power. The probability of detection as a function of SNR curves is drawn considering 500 radar pulses.

Figure 4a shows that the proposed max-SINR joint precoder–decoder design leads to a significant performance gain in target detection because interference from the communication system is almost completely eliminated. Even in a low-SNR regime where the SNR is below 0 dB, the proposed design still provides some performance gain in target detection. The SSSVSP method is not able to suppress interferences caused at communication transmitters due to the limitations of the precoder-only design.

Consequently, the performance of the radar subsystem is severely degraded, as shown by the probability of detection as a function of SNR in Figure 4b, where the radar signal is overwhelmed by interference such that the target cannot be reliably detected using SSSVSP. Increased signal power at the transmitters will further reduce the performance.

**Figure 5:**
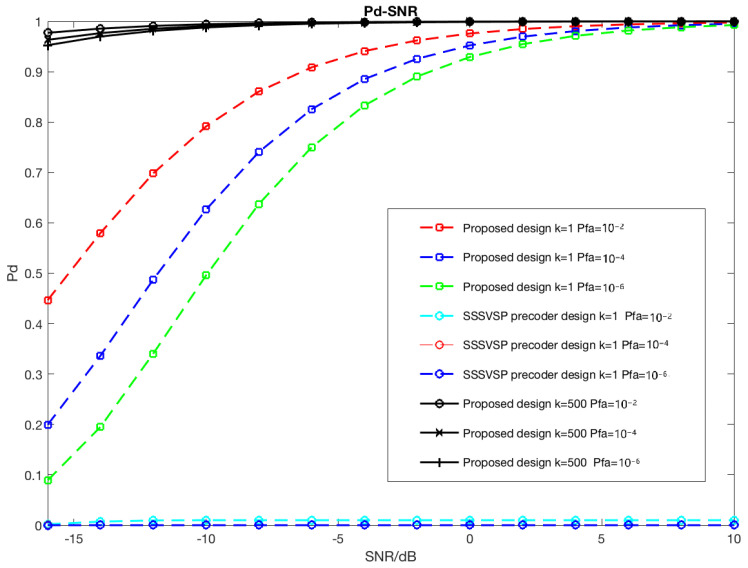
Detection performance difference comparison between proposed max-SINR joint precoder–decoder design and SSSVSP at $P_{fa}=10^{-2},P_{fa}=10^{-4}$, and $P_{fa}=10^{-6}$. A single pulse k=1 and multi-pulse k=500 are considered in this comparison.

In Figure 5, the difference in radar performance between the proposed max-SNR joint precoder–decoder design and SSSVSP is shown. A Neyman–Pearson detector is applied with false alarm constraints $P_{fa}=10^{-2},P_{fa}=10^{-4}$, and $P_{fa}=10^{-6}$.

The performances of pulse numbers k=1 and 500 are also compared in this simulation as a function of the number of pulses. At this time, the SINR value of SSSVSP is always below 0 dB. However, by using the proposed precoder–decoder with IA, the detectors achieve $P_{d}=1$ at a much lower SNR than by any other method. By increasing the number of pulses, the radar detection performance improves because the radar performance benefits from coherent signal processing. A large gap in performance between the proposed design and SSSVSP is observed. In the proposed design, the radar detection probability is above 0.85 when the SNR is above 0.

**Figure 6:**
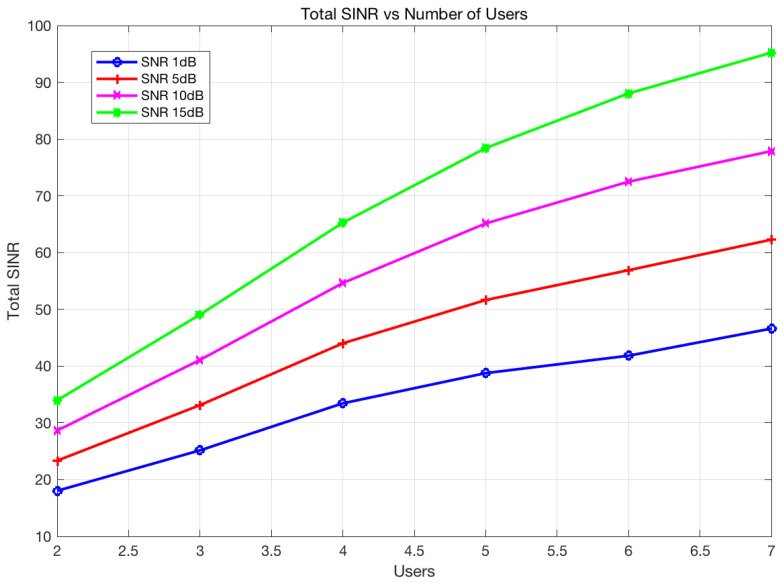
System SINR performance comparison as a function of the number of users for the proposed design. The user-desired DoF is 1.

In Figure 6, the SINR performance is studied as a function of the number of users. In this simulation, the DoF of each user is 1. The results show that total system SINR increases as the number of users increases. An additional user may add a useful signal to the system. However, it will cause interference for the other users. The proposed design can successfully remove interference for the entire system. The higher SNR at each receiver could also increase the system SINR. The proposed design avoids performance loss when the number of users increases.

## 5. Conclusions

This paper considered the problem of interference management and alignment in radar and communication coexistence and spectrum sharing scenarios. A generalized multicarrier signal model for coexistence was proposed. The model provides an easy way to model, generate and analyze a multicarrier signal. A max-SINR joint precoder–decoder joint design based on IA theory was proposed. The design was formulated as a constrained optimization problem. By employing IA theory, the benefit of achieving the total DoF upper bound can be obtained. A distributed algorithm for finding the precoder and decoder was derived as a solution to a constrained optimization problem. This takes advantage of channel reciprocity.

The proposed design allows for achieving better interference management between communication and radar nodes in comparison to the SSSVSP precoder design. The simulation results demonstrated that our algorithm significantly improved the total SINR for all radar and communication nodes and provided a significantly higher target-detection probability in a radar subsystem at a given constraint on a false alarm probability level while making the communication system almost interference free. The total SINR increased as a function of the SNR and the number of users while avoiding performance degradation.

## Data Availability

Not applicable.

## Abbreviations

The following symbols are used in this manuscript:

α	scalar, complex path loss
A	transmitted signal matrix under block fading assumption
A	communication user set
$A_{r}\;\;\;$	communication user set that is interfered by radar
$A_{c}\;\;\;$	communication user set that is not interfered by radar
β	baseband subcarrier
$\beta_{R}\;\;\;$	radar baseband subcarrier
$\beta_{C}\;\;\;$	communication baseband subcarrier
B	division multiplexing modulation matrix
$\ddot{B}\;\;\;$	demodulation matrix
B	radar user set
C	channel coding matrix
d	scalar, number of degrees of freedom
D	interference plus noise covariance matrix
$h[f_{1}]\;\;\;$	scalar, channel impulse response received for first subcarrier
H	channel matrix
K	total number of communication users
L	total number of blocks/pulses
M	total number of subpulses
N	column dimension of precoder matrix, which is user-desired frequency DoFs.
$N_{sc}\;\;\;$	number of transmitted subcarriers
$N_{p}\;\;\;$	column dimension of data matrix S
P	$N_{sc}\times N$-dimensional precoding matrix
Q	$N_{sc}\times N$-dimensional decoding matrix
s(N)	data vector for *N*th time slot
S	$N_{p}\times M$-dimensional data matrix
$T_{p}\;\;\;$	entire pulse duration
W	$N_{sc}\times M$-dimensional Gaussian noise matrix
$Y_{T}\;\;\;$	$N_{sc}\times LM$-dimensional transmitted signal matrix
$Y_{R}\;\;\;$	$N_{sc}\times LM$-dimensional received signal matrix
$\tau_{T,i}\;\;\;$	scalar, delay between transmitted antenna and target
$\tau_{R,i}\;\;\;$	scalar, delay between received antenna and target
θ	target’s direction of arrival
Ω	selection matrix
$1_{N\times M}\;\;\;$	N×M-dimensional all-ones matrix

## Appendix A

A multicarrier radar and communication signal model with $N_{sc}$ available subcarriers may be described as in (A1). The coded data/waveforms are mapped to subcarriers after precoding and channel coding. Pulse multicarrier radar signals and continuous multicarrier communication signals are employed in this model.

(A1)
$$Y_{T}\left(k\right)=\left\{\begin{array}{cc} 2Re\{\sum_{x=0}^{N_{sc}-1}\sum_{y=0}^{N_{p}-1}\sum_{m=0}^{M-1}\sum_{n=0}^{N-1}p_{k,n}c_{n,y}s_{y,m}\;rect\left[\frac{kT_{c}}{N_{sc}}-\left(x+am\right)T_{c}\right]\;\times e^{j2\pi \left(f_{c}+x\Delta f\right)\frac{kT_{c}}{N_{sc}}}\;\} & \mathrm{for}\;\mathrm{radar}\;\\ 2Re\{\sum_{x=0}^{N_{sc}-1}\sum_{y=0}^{N_{p}-1}\sum_{m=0}^{M-1}\sum_{n=0}^{N-1}p_{k,n}c_{n,y}s_{y,m}\;rect\left[\frac{kT_{c}}{N_{sc}}-xT_{c}\right]\;e^{j2\pi \left(f_{c}+x\Delta f\right)\frac{kT_{c}}{N_{sc}}}\}, & \mathrm{for}\;\mathrm{communication}\; \end{array}\right.$$

A rectangular pulse shape function is denoted by rect(t) in (A1). $Y_{T}\left(k\right)$ is an element of matrix $Y_{T}$. The remaining notations are defined as follows:

- m={0,1,2,⋯,M−1} is the index of subpulses, where *M* is the number of subpulses.
- n={0,1,2,⋯,N−1} is the index of channel coded data sequence, where *N* is the number of channel coded data symbols; it essentially defines user-desired DoFs.
- $y=\{0,1,2,\cdots ,N_{p}-1\}$ is the index of data sequence before channel coding, where $N_{p}$ is the number of original, uncoded data symbols.
- $x=\{0,1,2,\cdots ,N_{sc}-1\}$ denotes the index of subcarriers, where $N_{sc}$ is the total number of transmitted subcarriers.
- $k=\{0,1,2,\cdots ,MN_{sc}-1\}$ denotes the sample index.
- *a* is a multiplicity factor between communication symbol duration $T_{c}$ and radar subpulse duration $T_{p}$, where $T_{p}$ is larger than $T_{c}$ considering zero-padding in pulse radar. Then, the radar subpulse duration is given by $T_{p}=aT_{c}$.
- $c_{n,y}$ is a scalar with respect to the *n*th subcarrier and *y*th data symbol, $c_{n,y}$ denotes the coding operator for radar signal and the channel coding operator in the communication signal; e.g., it will be exp(jϕ) for P3 or P4 radar code and an element of the Turbo generator matrix for Turbo code.
- $s_{y,m}$ is scalar data corresponding to the *y*th data symbol and *m*th subpulse, which is random for the payload communication signal and known in radar applications because the transmitted waveform information is known by its receiver.
- $p_{k,n}$ is a scalar that denotes the precoding coefficient with respect to the *k*th subcarrier and *n*th subpulse before the signal emitted.

A detailed derivation of (A1) is given in the following. Considering an OFDM signal with $N_{sc}$ subcarriers and Δf Hz bandwidth, the continuous-time complex envelope of the baseband OFDM signal can be written for any instant *t* as

(A2) $$s_{m}\left(t\right)=\sum_{x=0}^{N_{sc}-1}w_{m,x}\;rect\left[t-xT_{c}\right]\;e^{j2\pi x\Delta ft}$$

where rect[·] is a discrete rectangular window, $T_{c}$ is the symbol duration, and the constraint $T_{c}=\frac{1}{\Delta f}$ satisfies the spectrum selection channel condition. $w_{x}$ indicates the amplitude weighting of the *x*th subcarrier. Additionally,

(A3) $$rect\left(t\right)=\left\{\begin{array}{cc} 1 & \mathrm{if}\;\;0\leq t\leq T_{c},\\ 0 & \mathrm{otherwise}. \end{array}\right.$$

The broadcast multicarrier communication signal is considered to be continuous:

(A4) $$y_{T,c}\left(t\right)=\sum_{m=-\infty }^{\infty }s_{m}\left(t-mT_{c}\right).$$

In general, a multicarrier radar signal can be written as follows:

(A5) $$y_{T,r}\left(t\right)=\sum_{m=0}^{M-1}s_{m}\left(t-mT_{p}\right),$$

where *M* is the number of OFDM subpulses, $T_{p}=aT_{c}$, $a\in R^{+}$ is the radar pulse duration. As both radar and communication signals coexist, are designed jointly and are postprocessed simultaneously in this paper, time synchronization is employed. The transmitted signal is a real part of the baseband complex envelope; then, (A5) can be rewritten as follows:

(A6) $$\begin{array}{cc} {\hat{y}}_{T,r}\left(t\right) & =2Re\{\sum_{m=0}^{M-1}s_{m}\left(t-mT_{p}\right)\;\}\\ \; & =2Re\{\sum_{m=0}^{M-1}\sum_{x=0}^{N_{sc}-1}w_{m,x}\;rect\left[t-xT_{c}-mT_{p}\right]\;\}\\ \; & e^{j2\pi x\Delta ft}\;\}\\ \; & =2Re\{\sum_{m=0}^{M-1}\sum_{x=0}^{N_{sc}-1}w_{m,x}\;rect\left[t-\left(x+am\right)T_{c}\right]\;\\ \; & e^{j2\pi x\Delta ft}\;\} \end{array}$$

According to the Nyquist–Shannon sampling theorem, the sampling rate is $N_{sc}\Delta f$ samples/second. Additionally, replacing $w_{m,x}$ with $p_{k,n}$, $c_{n,y}$ and $s_{y,m}$, the radar transmitted signal can be rewritten as follows:

(A7) $$\begin{array}{cc} {\hat{y}}_{T,r}\left(k\right)= & 2Re\{\sum_{x=0}^{N_{sc}-1}\sum_{y=0}^{N_{p}-1}\sum_{m=0}^{M-1}\sum_{n=0}^{N-1}p_{k,n}c_{n,y}s_{y,m}\;\\ \; & rect\left[\frac{kT_{c}}{N_{sc}}-\left(x+am\right)T_{c}\right]\;\times e^{j2\pi \left(f_{c}+x\Delta f\right)\frac{kT_{c}}{N_{sc}}}\} \end{array}$$

The transmitted communication signal can also easily be derived.

## Appendix B

From (5), we define $Y_{T}=\left(\Omega \circ B\right)PCS$ as a compact matrix form of transmitted signal. The received response in the time domain is

(A8) $$\begin{array}{c} Y_{R}=\ddot{B}G\left(\theta \right)Y_{T} \end{array}$$

where $\ddot{B}$ is the demodulation matrix, $\ddot{B}B=I_{N_{sc}\times N_{sc}}$. Considering that the channel coefficients are $h_{i}\left(\theta \right),i=1,\cdots ,N_{sc}$, a guard interval (or cyclic prefix) with length $N_{sc}$ is employed. Note that the signal is transformed into the frequency domain after modulation. To complete the convolution through matrix multiplication, G(θ) is defined as a circulant matrix that is given by the following $N_{sc}$ by $N_{sc}$ matrix [66],

(A9) $$G\left(\theta \right)=\left(\begin{array}{cccc} h_{1}\left(\theta \right) & h_{2}\left(\theta \right) & \cdots & h_{N_{sc}}\left(\theta \right)\\ h_{N_{sc}}\left(\theta \right) & h_{1}\left(\theta \right) & \cdots & h_{N_{sc}-1}\left(\theta \right)\\ \cdots & \cdots & \cdots & \cdots \\ h_{2}\left(\theta \right) & h_{3}\left(\theta \right) & \cdots & h_{1}\left(\theta \right) \end{array}\right)$$

The circulant matrix G(θ) can be diagonalized by eigenvalue decomposition. In the OFDM case, G(θ) can be written as follows:

(A10) $$G\left(\theta \right)=B^{H}H\left(\theta \right)B.$$

where $H(\theta )=\mathrm{diag}\{h_{1}\left(\theta \right),h_{2}\left(\theta \right)\cdots ,h_{N_{sc}}\left(\theta \right)\}$ is a diagonal eigenvalue matrix. Matrix B is symmetric and unitary for the OFDM signal, $\ddot{B}=B^{H}=B$. Then, the received signal is given by the following:

(A11) $$\begin{array}{cc} Y_{R} & =\ddot{B}G\left(\theta \right)Y_{T}\\ \; & =B^{H}B^{H}H\left(\theta \right)BB\circ \Omega PCS\\ \; & =H(\theta )(\Omega \circ I)PCS \end{array}$$
